# US-localized diffuse optical tomography in breast cancer: comparison with pharmacokinetic parameters of DCE-MRI and with pathologic biomarkers

**DOI:** 10.1186/s12885-016-2086-7

**Published:** 2016-02-01

**Authors:** Min Jung Kim, Min-Ying Su, Hon J Yu, Jeon-Hor Chen, Eun-Kyung Kim, Hee Jung Moon, Ji Soo Choi

**Affiliations:** Department of Radiology, Breast Cancer Clinic, Severance Hospital, Research Institute of Radiological Science, Yonsei University College of Medicine, Seoul, South Korea; Department of Radiological Sciences, University of California, Irvine, CA USA; Department of Radiology, Eda Hospital and I-Shou University, Kaohsiung, Taiwan; Department of Radiology, Samsung Medical Center, Seoul, Korea

## Abstract

**Background:**

To correlate parameters of Ultrasonography-guided Diffuse optical tomography (US-DOT) with pharmacokinetic features of Dynamic contrast-enhanced (DCE)-MRI and pathologic markers of breast cancer.

**Methods:**

Our institutional review board approved this retrospective study and waived the requirement for informed consent. Thirty seven breast cancer patients received US-DOT and DCE-MRI with less than two weeks in between imaging sessions. The maximal total hemoglobin concentration (THC) measured by US-DOT was correlated with DCE-MRI pharmacokinetic parameters, which included K^*trans*^, k_*ep*_ and signal enhancement ratio (SER). These imaging parameters were also correlated with the pathologic biomarkers of breast cancer.

**Results:**

The parameters THC and SER showed marginal positive correlation (r = 0.303, *p* = 0.058). Tumors with high histological grade, negative ER, and higher Ki-67 expression ≥20 % showed statistically higher THC values compared to their counterparts (*p* = 0.019, 0.041, and 0.023 respectively). Triple-negative (TN) breast cancers showed statistically higher K^*trans*^ values than non-TN cancers (*p* = 0.048).

**Conclusion:**

THC obtained from US-DOT and K^*trans*^ obtained from DCE-MRI were associated with biomarkers indicative of a higher aggressiveness in breast cancer. Although US-DOT and DCE-MRI both measured the vascular properties of breast cancer, parameters from the two imaging modalities showed a weak association presumably due to their different contrast mechanisms and depth sensitivities.

## Background

Mammography is a sensitive imaging method for detection of breast cancers [1] and that has contributed to the improvement of the survival rates for breast cancer [2]. However, the sensitivity of mammography drops down to 62 % in cases of dense breasts [3]. Complementary imaging methods have been introduced to identify mammographically occult breast cancers, as well as to differentiate malignant lesions from benign lesions based on the morphologic and physiologic characteristics of breast lesions [4–14]. Ultrasonography (US) is the most commonly used supplemental imaging method to improve the sensitivity of breast cancer detection; however, it is also known to yield a high number of false positives [4, 6, 12]. Several additional techniques, including elastography, Doppler, and optical imaging, have been introduced to improve the specificity of US through leveraging functional parameters that complement the traditional morphological parameters [7, 10, 15].

Diffuse optical tomography (DOT) is a suitable breast imaging modality that measures functional characteristics of breast lesions, by using near infrared light to probe tissue optical properties. The parameters that can be measured include the concentrations of water, lipid, as well as oxy-hemoglobin and deoxy-hemoglobin that can be used to calculate the total hemoglobin concentration and the oxygen saturation. Hemoglobin concentration is known to be related to angiogenesis, which is critical for autonomous growth and the spread of breast cancer [16, 17]. However, the low spatial resolution of DOT has limited its clinical application [18]. Recently, the availability of ultrasonography-guided diffuse optical tomography (US-DOT) has increased its usefulness as a complementary imaging modality for breast imaging, with the technique combining both morphologic characteristics found with US and functional information found with DOT [15, 19, 20]. In a previous report on patients with breast cancer, the total hemoglobin measured by US-DOT was correlated with tumor size and several molecular biomarkers (HER2 and Ki-67), and it was shown to have potential for predicting tumor aggressiveness [21].

Another approach to measure angiogenic properties of breast tissue is dynamic contrast-enhanced MRI (DCE-MRI), an important clinical imaging modality for detection and diagnosis of breast cancer. In addition to providing high quality breast images not limited by dense breasts, it can also be used to access vascular information by using a dynamic imaging protocol. Pharmacokinetic parameters such as K^*trans*^ and k_*ep*_ are commonly used to characterize neovascularization in breast cancer. These kinetic parameters are also reported to correlate with biomarkers and can be used to predict poor prognosis [22]. Therefore, both US-DOT and DCE-MRI can be applied to measure tumor angiogenesis, and are known to yield quantitative parameters for characterizing angiogenic properties of tumors. However, there have been few studies that compare US-DOT and DCE-MRI to evaluate their correlation.

The purpose of this study was to investigate the correlation of parameters measured by US-DOT with pharmacokinetic features measured by DCE-MRI to evaluate breast tumor angiogenesis, as well as to investigate the association of these imaging parameters with pathologic and molecular biomarkers of breast cancer.

## Methods

This study was approved by the Severance Hospital Institutional Review Board, and the requirement for informed consent was waived for this retrospective study. Patients gave informed consent prospectively prior to US-DOT when they were diagnosed with breast cancer, and the written informed consent included consent for the future use of their US-DOT information in the comprehensive research of breast disease.

### Study population

Among 63 consecutive pathologically-proven breast cancer patients who underwent US-DOT between June 2009 and August 2009 in our institution, 37 patients with breast cancer underwent diagnostic breast DCE-MRI within 2 weeks of US-DOT imaging. All of these patients underwent surgery at our institution and were included in the analysis for this study. Because core-biopsy can affect the value of US-DOT parameters, US-DOT imaging was done before the core-biopsy for all cases in our study.

### US-localized diffuse optical tomography

The US-DOT was done using a commercially available breast diagnostic equipment, OPTIMUS type II (XinaoMDT Technology Co., Ltd, China). It is a dual imaging modality combining conventional ultrasound (Terason T3000 ultrasound, Teratech, USA) and near-infrared (NIR) optical tomography, which can be used to measure functional tissue properties with optical spectroscopic analysis. The main functional parameter is the oxy- and deoxy-hemoglobin concentration calculated from absorption coefficients measured by using two optical wavelengths (785 nm and 830 nm). The optical probe delivered light with an array of nine optical fibers and detected reflected light through the tissue with an array of ten optical guides [23]. The technical details of this imaging system, including system configurations, imaging acquisition methods, and the data processing algorithms, have been described in a previous report [15]. The US-DOT system can detect up to 35 mm into the tissue. The system reconstructs 7 slices from the skin, each with 5 mm thickness. For the thirty seven patients in our study, the mean size of breast lesions was 18.4 mm. We carefully positioned the breast of each subject to ensure that US-DOT could cover the entire lesion. The mean thickness of the breast, defined as the distance between the skin surface to the chest wall muscle, was 20.9 mm on US image. With the exception of 2 cases, the breast thickness was smaller than 25 mm. After conventional US evaluation, the US-DOT imaging procedure was done using the hybrid handheld probe through following the manufacturer’s recommended protocol. Briefly, the lesion was identified by a linear 7–12 MHz ultrasound transducer in the center of the hybrid probe to find the maximal diameter of the lesion.

**Fig. 1:**
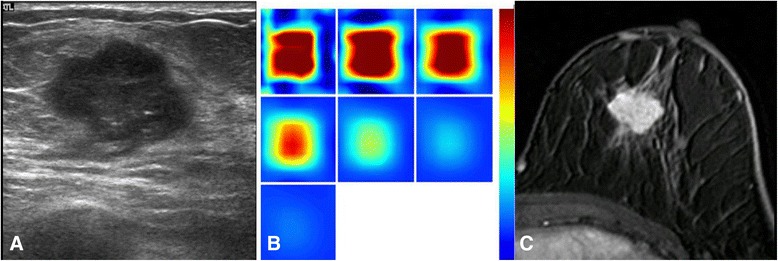
A woman with invasive ductal carcinoma (**a**) A gray-scale ultrasound image shows a hypoechoic mass with microlobulated margins, measuring 1.8 cm in diameter (high histologic grade, LVI (−), ER(−), PR(−), HER-2 (+), Ki-67 (+)). **b** A reconstructed optical absorption map shows a distinct mass with a high maximum THC of 293.4 μmol/L. The first section (slice 1, top left) is a 6 × 6 cm spatial x-y image (coronal plane of the body) obtained at a depth of 0.5 cm, as measured from the skin surface. The last section (slice 7, bottom left) is a 6 × 6 cm spatial x-y image (coronal plane of the body) obtained at a depth of 3.5 cm, as measured from the skin surface. Spacing between sections is 0.5 cm in the direction of propagation. **c** A lobular homogenously enhancing mass is noted from one of the DCE-MRI slices. The *K* ^trans^ is 0.122 [1/min], the *k*_ep_ is 0.415 [1/min] and the SER is 1.024

Based on the US images, a square region of interest (ROI) was drawn to include the maximal diameter and the perpendicular dimension of the lesion. Since the ROI was a square shape, it encompassed the whole area of the identified lesion and a small portion of the surrounding normal tissues. Then the optical imaging was acquired using the same hybrid probe. The plane that showed the maximal diameter of the tumor was used as the optical horizontal plane. Then, the probe was rotated by 90° angle to acquire the optical data from the vertical plane. Next, we performed the same process in the symmetric region in the contralateral normal breast, including the horizontal and vertical planes. The optical imaging measured the normal site in the symmetrical region of the contralateral breast was used as references in the reconstruction. After scanning the four planes (two lesion planes and two contralateral normal planes), the optical characteristic parameters and the total hemoglobin concentration (THC, micromoles per liter) were obtained by calculating the difference between the lesion and the symmetric normal site, and the images were displayed on the screen of the imaging system. The maximal THC value was determined as the maximal hemoglobin concentration in the region of interest box (Fig. 1a , b).

### DCE-MRI study protocol

Breast MR imaging was performed with a patient in the prone position using a 1.5 T MR scanner (Philips Healthcare, Best, Netherlands) with a dedicated bilateral breast coil. The DCE-MRI sequence was based on a 3D gradient echo sequence (repetition time/echo time, 7.0/3.4 ms; flip angle, 12°; bandwidth 215 Hz/pixel; slice thickness, 3 mm; FOV, 340 mm × 340 mm; matrix size, 368 × 302; voxel size, 0.7 × 0.7 × 3.0 mm) with axial sections. A total of 7 dynamic frames (repetitions) were acquired. Each frame took 66 s resulting in a total imaging time of approximately 7 min and 42 s. Gadolinium diethylene triaminepenta acetic acid (Gd-DTPA, Magnevist; Berlex Laboratories, Inc., Montville, NJ, USA; 0.2 cc/kg) was injected manually at the start of the second-frame acquisition, and then followed by a 10-cc saline flush. The total injection time of the contrast agent was maintained between 15 and 20 s for every patient to make the bolus length as consistent as possible. The saline flush was given as a fast bolus. All MR images were transferred from the MR-console to a personal computer for post-processing.

### DCE-MRI kinetic parameters

The analysis of DCE-MRI enhancement kinetics was done by a radiologist with 8 years of experience in breast imaging interpretation. The tumor was determined from the color-coded enhancement maps which were generated by subtracting the pre-contrast images from the first post-contrast images. On each imaging slice showing the enhanced tumor, a ROI was manually drawn to outline the entire tumor (e.g. Fig. 1c). The signal intensity time course was calculated from each ROI, and the calculated time courses from all the tumor ROIs drawn on different imaging slices were averaged to calculate a mean signal intensity time course for this study. Signal intensities measured from seven post-contrast frames were normalized by the signal intensity measured from the pre-contrast images. The enhancement kinetics was then analyzed by using the Tofts two-compartmental pharmacokinetic model [24]. The pharmacokinetic parameters, K^*trans*^, and k_*ep*_, represented the uptake rate and washout rate of the Gadolinium contrast agent, respectively. A Matlab program (version 6.0.0.88; The MathWorks, Inc., USA) was written to fit the measured enhancement time course to the time course generated by the two-compartmental model, and the parameters K^*trans*^, and k_*ep*_ were obtained after the fitting. The signal enhancement ratio (SER) was related to the washout slope in DCE kinetics and calculated as: SER = (S1-S0)/(S2-S0), where S0 is the pre-contrast signal intensity, S1 is the peak signal intensity approximately at 90 s post injection, and S2 is the signal intensity at the last time point in the DCE sequence.

### Pathologic parameters

Histopathological results and molecular biomarkers, including tumor size, histologic grade (HG), estrogen receptor (ER), progesterone receptor (PR), HER-2, Ki-67, lymphovascular invasion and axillary lymph node metastasis (LN mets), were evaluated for each case from surgical specimen. Histologic grade was determined with evaluation of mitosis, tubular formation and nuclear grade, all correlated with cellularity. The status of ER, PR, HER-2 and Ki-67 were determined based on pathologic results with immunohistochemical assays. Tumors with ≥ 1 % nuclear-stained cells were considered positive for ER and PR according to the American Society of Clinical Oncology/College of American Pathologists (ASCO/CAP) guidelines. HER-2 was considered positive for 3 +, or 2+ with amplification on the FISH test. Triple-negative breast cancer (TNBC) was defined as breast cancers showing negative ER, PR, and HER-2. Ki-67 staining was assessed with the percentage of nuclei showing a positive reaction. An arbitrary cut-off point of ≥ 20 % was used to define high Ki-67 expression, while the value less than 20 % was low. The tumor size was determined as the maximal diameter of the invasive component at surgical pathology. The presence of axillary lymph node was determined with surgical pathologic reports; and the presence of systemic metastasis was determined with medical records.

The more aggressive tumor was defined by larger tumor size, high histologic grade, negative ER, TNBC, high Ki-67 expression, positive lymphovascular invasion, and the presence of positive axillary lymph node metastasis and systemic metastasis.

### Statistical analysis

Pearson correlation was employed to determine whether the THC and DCE-MRI kinetic parameters (K^*trans*^, k_*ep*_ and SER) were correlated with each other. In Pearson’s correlation, a coefficient |r| < 0.2 indicates a correlation that is very weak, 0.2 ≤ |r| < 0.4 weak, 0.4 ≤ |r| ≤ 0.6 moderate, 0.6 ≤ |r| < 0.8 strong, and |r| ≥ 0.8 very strong [25]. The lesions were separated into two dichotomized groups based on each pathologic biomarker, and the difference between the values of imaging parameters in the two groups was compared using the student *t*-test. Statistical analysis was performed using the SPSS statistical analysis software (IBM SPSS Statistics, version 20.0.0; SPSS, Chicago, Ill), with the significance level set at a two-sided *p* value of < 0.05.

## Results

All 37 patients underwent surgery, and Table 1 shows the pathologic findings.

**Table 1:** Clinicopathologic biomarkers of the 37 breast cancer patients

Pathologic biomarkers		Number
Menstrual status	Premenopause	16
	menopause	21
Histology	Invasive ductal carcinoma	31
	Invasive lobular carcinoma	1
	Invasive micropapillary carcinoma	3
	Poorly differentiated carcinoma	2
Tumor size	<2 cm	20
	≥2 cm	17
Histologic grade	Low	16
	High	21
Estrogen receptor	Negative	15
	Positive	22
Progesterone receptor	Negative	13
	Positive	24
HER-2	Negative	31
	Positive	6
Triple-negative	Negative	27
	Positive	10
Ki-67	Low	18
	High	19
Lymphovascular invasion	Negative	23
	Positive	3
	Not available	11
Lymph node metastasis	Negative	24
	Positive	13

### The correlation between US-DOT parameter and DCE-MRI parameters

**Fig. 2:**
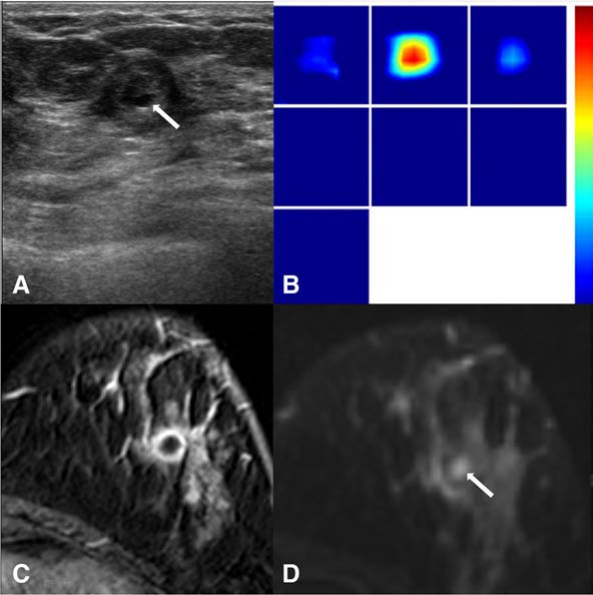
A woman with invasive ductal carcinoma (**a**) A gray-scale ultrasound image shows an isoechoic mass with central markedly hypoechoic component (arrow), which can present central necrosis (high histologic grade, LVI (−), ER(−), PR(−), HER-2 (−), Ki-67 (+)). **b** A reconstructed optical absorption map shows a distinct mass with a high maximum THC of 377.3 μmol/L with central prominent high signal. **c** dynamic contrast-enhanced MRI shows a round mass with rim enhancement with central non-enhancing area, correlated with central hypoechogenicity on US (**a**) and high signal intensity (arrow) on T2 weighted image (**d**). The *K* ^trans^ is 0.132 [1/min], the *k*_ep_ is 0.521 [1/min] and the SER is 1.002. The surgical specimen shows central necrosis

Between the THC and DCE-MRI parameters, only THC and SER showed a weak correlation with statistically marginal significance (r = 0.303, *p* = 0.058, Table 2). A higher total hemoglobin concentration was correlated with a more rapid washout rate (Fig. 1). There was no statistical significance in the correlation of THC and other two DCE-MRI parameters (r = −0.237 with K^*trans*^_*,*_*p* = 0.157; r = −0.218 with k_*ep*_, *p* = 0.195). Fig. 2 illustrates one example of discordant findings between DCE-MRI and THC; while an unenhanced necrotic core is clearly noted on MRI, a high homogeneous THC map is shown on US-DOT. The mean and standard deviation value for each parameter are shown in Table 3.

**Table 2:** The correlation between US-DOT parameters and DCE-MRI kinetics in the 37 breast cancers

Pearson Correlation	THC	*P*
K^*trans*^	-.237	.157
k_*ep*_	-.218	.195
SER	.303	.058

**Table 3:** The mean and standard deviation of US-DOT and DCE-MRI parameters in the 37 breast cancers

		Mean ± Stdev
US-DOT	THC	181.296 ± 76.888
DCE-MR	K^*trans*^	0.133 ± 0.066
	k_*ep*_	0.482 ± 0.157
	SER	1.057 ± 0.063

### The correlation between US-DOT parameter and pathologic parameters

High histologic grade, ER-negativity, and higher Ki-67 expression ≥20 % showed a higher THC value with statistical significance (*p* = 0.019, 0.041, and 0.030, respectively) compared to their counterparts (Table 4). Since cancers with high-grade, negative ER and high Ki-67 were considered as more aggressive, THC was associated with aggressiveness.

**Table 4:** Total hemoglobin concentration of US-DOT according to pathologic biomarkers

	Total hemoglobin concentration (μmol/L)		
		Mean	*p*
Tumor size	<2 cm (*n* = 20)	172.15	0.440
	≥2 cm (*n* = 17)	192.06	
HG	Low (*n* = 16)	147.98	0.019
	High (*n* = 21)	206.68	
ER	Neg (*n* = 15)	212.27	0.041
	Pos (*n* = 22)	160.02	
PR	Neg (*n* = 13)	211.86	0.075
	Pos (*n* = 24)	164.73	
HER-2	Neg (*n* = 31)	179.63	0.769
	Pos (*n* = 6)	189.91	
TNBC	Neg (*n* = 27)	173.12	0.294
	Pos (*n* = 10)	203.38	
Ki-67	Low (*n* = 18)	153.52	0.030
	High (*n* = 19)	207.61	
LVI	Neg (*n* = 23)	175.45	0.581
	Pos (*n* = 3)	146.14	
LN mets	Neg (*n* = 24)	179.84	0.879
	Pos (*n* = 13)	183.97	
mets	Neg (*n* = 36)	180.36	0.663
	Pos (*n* = 1)	214.97	

### The correlation between DCE-MRI parameters and pathologic parameters

For DCE pharmacokinetic parameters, triple-negative (TN) breast cancers showed a higher K^*trans*^ than non-TN tumors (*p* = 0.048). Cases with negative HER-2 had higher K^*trans*^ values than those with positive HER-2; and cases with high Ki-67 ≥ 20 % had higher K^*trans*^ than those with Ki-67 < 20 %, with marginally statistical significances (*p* = 0.051 and 0.060, respectively, Table 5). There was no significant difference in *k*_*ep*_ between tumors with different pathologic parameters or molecular biomarkers.

**Table 5:** Parameters of DCE-MRI according to pathologic biomarkers

		K^trans^		k_ep_		SER	
		(1/min)		(1/min)			
		Mean	*p*	Mean	*p*	Mean	*p*
Overall							
Tumor size	<2 cm (*n* = 20)	0.134	0.885	0.495	0.440	1.118	0.471
	≥2 cm (*n* = 17)	0.131		0.468		1.085	
HG	Low (*n* = 16)	0.136	0.829	0.484	0.965	1.136	0.199
	High (*n* = 21)	0.131		0.481		1.077	
ER	Neg (*n* = 15)	0.156	0.124	0.513	0.337	1.111	0.746
	Pos (*n* = 22)	0.118		0.462		1.096	
PR	Neg (*n* = 13)	0.154	0.162	0.485	0.932	1.119	0.613
	Pos (*n* = 24)	0.122		0.481		1.091	
HER-2	Neg (*n* = 31)	0.138	0.051	0.487	0.684	1.109	0.526
	Pos (*n* = 6)	0.107		0.458		1.069	
TNBC	Neg (*n* = 27)	0.116	0.048	0.482	0.985	1.103	0.996
	Pos (*n* = 10)	0.178		0.483		1.103	
Ki-67	Low (*n* = 18)	0.113	0.060	0.470	0.629	1.103	0.989
	High (*n* = 19)	0.154		0.495		1.102	
LVI	Neg (*n* = 23)	0.135	0.514	0.504	0.818	1.115	0.726
	Pos (*n* = 3)	0.113		0.478		1.082	
LN mets	Neg (*n* = 24)	0.123	0.206	0.498	0.412	1.112	0.581
	Pos (*n* = 13)	0.152		0.453		1.085	

## Discussion

As tumors cannot grow beyond 2 mm simply with nutrients supplied through diffusion, angiogenesis becomes a critical process for sustained tumor growth. Angiogenesis is capable of differentiating between malignant and benign tumors and can be used as a discriminating characteristic of aggressiveness [16]. The wall of neovascularity tends to be leaky, and the increased permeability results in early and rapid contrast-enhancement on MRI. Pharmacokinetic parameters are very useful in the characterization of angiogenesis in breast cancer and have been shown to be associated with the spread of breast cancer and patient prognosis.

The THC measured by DOT represents blood volume, which has been reported to have high values in malignant tumors [15, 20, 26]. Therefore, high THC measured by DOT is generally associated with tumors showing contrast-enhancement identified by MRI [27–30]. Several MR-compatible DOT systems have been developed for breast imaging, which has the advantage of improving the quality of reconstructed DOT images by using the morphological information provided by DCE-MRI as *a priori* information [28–33]. Since tumors were co-registered, the obtained information by DCE-MRI and DOT could be easily compared. The suspicious malignant lesions on DCE-MRI were reported to show higher mean absorption coefficient than benign lesions [32].

DCE-MRI is an established clinical imaging modality for breast cancer. For research, pharmacokinetic analysis is commonly applied to obtain parameters. K^*trans*^ is the inflow transfer constant, which is related to the delivery of contrast agent to the tumor through vascular perfusion and permeability, while k_*ep*_ is the out-flux transfer rate constant for the contrast agent to diffuse from the extracelluar extravascular space back to the plasma compartment [26]. The signal enhancement ratio measures the washout slope based on signal intensities at three time points, which is also related to perfusion and permeability [34]. These DCE-MRI parameters as well as the THC measured by DOT have been correlated with the histologic microvascular density count [35].

The mean THC results could be affected by tumor heterogeneity and the partial volume effect (i.e. inclusion of normal issues in the measurement) [36]; therefore, in this study we chose to analyze the maximal THC, which was measured as the maximum THC value within the tumor ROI box. A suggested cutoff value of THC for malignancy was 140 μmol/L in a previous report [20], but it was also reported that many malignant tumors could have a lower THC value around 100 μmol/L [36]. In our results, the mean value of the THC was 181.3 μmol/L, comparable with results in the previous report [20]. In the correlation analysis between US-DOT parameters and clinicopathologic characteristics, several poor prognostic biomarkers, including high histologic grade, ER negativity and high Ki-67 expression, were significantly correlated with a high THC. Histologic grade is one of three strongest prognostic determinants, which include LN mets, tumor size and histologic grade [37]. Ki-67 is a marker of cell proliferation including the S-phase fraction, mitotic index and bromodeoxyuridine uptake [38]. High Ki-67 expression has been regarded as a characteristic of more aggressive proliferation as well as neovascularization; it is also associated with a good chance of clinical response to chemotherapy [39, 40]. In this study, the THC in high Ki-67 ≥ 20 % cancers (mean ± SD, 207.61 ± 80.15 μmol/L) was higher than in low Ki-67 < 20 % cancers (mean ± SD, 153.52 ± 64.07 μmol/L). With our results, it could be suggested that breast cancers showing a high THC have poorer prognosis than those with a low THC. There was no difference between THC values in HER-2 positive and HER-2 negative groups (p > 0.05), different from Brown et al. [41] and Choi et al. [21]. In our study population, only 6 cancers were HER-2 positive while 31 were HER-2 negative. The low rate of HER-2 positive cancers was possibly from case selection bias (because both US-DOT and DCE-MRI scans were required), and the insufficient case number might affect the results. We also found a higher THC value in larger tumors (≥2 cm) than in smaller ones (<2 cm), but the difference was not statistically significant.

As the use of breast US-DOT in current clinical practice increases [15, 19, 21, 35, 36], there has been efforts to assess the correlation of THC with parameters measured by other imaging modalities [42, 43]. Zhu et al. compared US-DOT with the color Doppler flow imaging and found that the THC value did not differ significantly in malignancies with or without vascular tissue shown on Doppler flow imaging. Doppler flow imaging is based on detection of blood flow motion with relatively high velocity in large vessels, while optical imaging is mainly sensitive to the capillary blood volume within the tumor, which might explain the disagreement [43]. Similarly, in our study we did not find a high correlation between MR parameters and US-DOT results, presumably because of the different imaging principles on which the two techniques are based, as well as the analysis methods. Since a low molecular weight contrast agent (Gd-DTPA) is used for DCE-MRI, the agent can easily leak from the plasma compartment to the extravascular-extracellular compartment, and it is well known that the DCE kinetics are heavily dependent on the vascular permeability and the distribution volume in the extravascular-extracellular space [22, 26]. For example, DCE-MRI can miss breast cancers showing low angiogenesis such as in low-grade DCIS [44]. In contrast, the THC measured by optical imaging is mainly related to the total blood volume without involvement of vascular permeability or distribution space, therefore the fundamental differences in the contrast mechanism could explain the lack of a high correlation between DCE-MRI and US-DOT parameters.

Understanding the tumor microenvironment for angiogenesis can be complicated, and results obtained using different methods may not be well correlated. For example, although US-DOT, Color Doppler flow imaging, and DCE-MRI are all based vascular properties for measurements, Color Doppler imaging shows no significant correlation with microvessel counts [45], while some DCE-MRI parameters were reported to be correlated with microvessel density but not specifically with VEGF, a potent factor to stimulate angiogenesis [46]. Another major reason leading to the poor correlation of parameters is the high heterogeneous nature of the tumor. In this study, for DCE-MRI we included all enhanced tumor tissues from multiple imaging slices as ROI to evaluate kinetics on MR imaging, therefore, it is more like a “whole tumor analysis” approach. In US-DOT the maximum THC value in the ROI box was obtained and used for analysis, thus it is more like a “hot spot analysis” approach. Therefore, it is unlikely to have a high correlation between parameters obtained from “whole tumor” and “hot spot” analyses. However, it was not possible to do co-registered regional analysis due to the diffuse nature of the optical imaging. Optical imaging is very sensitive to the depth information, and tissues near the sensitive region of optical fibers will have more contribution to the measurement results. For example, as the case illustrated in Fig. 2, while DCE-MRI clearly shows a necrotic/cystic core, THC maps shows an averaged high blood volume, presumably due to the sensitivity to the strongly enhanced tissue near the surface closer to the source and detector fibers.

DCE-MRI parameters have been shown to be associated with poor prognostic factors such as high histologic grade and ER negativity [22]. Nevertheless, there have been inconsistent results due to different case numbers and the study population, e.g. Fernández-Guinea et al. [47]. In our study high Ki-67 expression and triple-negative breast cancers showed higher K^*trans*^ than their counterparts with marginal significance, suggesting that more aggressive tumors have a higher angiogenesis as measured by DCE-MRI. This result is consistent with a recent report which showed that the mean K^*trans*^ was higher in Ki-67-positive tumors than in Ki-67-negative tumors [48]. For further detailed analysis considering tumor heterogeneity, a histogram or pixel-by-pixel analysis can be considered [49]. However, this type of analysis is not meaningful in US-DOT due to the diffuse nature of the optical imaging methods.

There were some limitations in this study. First, the study population was limited to a small number of patients with malignant tumors who received both US-DOT and DCE-MRI. Since no benign tumors with lower angiogenesis were included in the analysis, the dynamic range was small and less likely to show a good correlation result as published in other studies using a diagnostic population. Second, the DCE-MRI was acquired using a typical clinical protocol with 7 dynamic frames and 66 s temporal resolution. This coarse temporal resolution was not sufficient to obtain vascular volume characteristics at a very early time after contrast injection, which is expected to have a better correlation with THC measured by US-DOT [50]. Also, we did not measure the pre-contrast T1 relaxation time T10, and could not measure the arterial input function from each individual patient. The T10 and the arterial input function may vary between patients, and if these parameters can be accurately measured from each patient and used in the pharmacokinetic model fitting, more precise K^trans^ and k_ep_ may be obtained. However, these measurements are difficult to do and not practical in a clinical setting; also variations in the resulting Ktrans and kep values are expected to be small and are not thought to affect the correlation with THC. Nonetheless, the pharmacokinetic analysis obtained using assumed T10 and the population blood curves is a common approach and can yield characteristic K^trans^ and k_ep_ that are highly correlated with parameters directly calculated from DCE kinetics. The DCE-MRI was done within 2 weeks after US-DOT. The vascularity of breast tissues is known to vary in different phases of a menstrual cycle; therefore, this may introduce a small variation in 16 pre-menopausal women [51]. However, the vascularity of the tumor is much higher compared to normal tissues, and it is unlikely to change much in 2 weeks. Since we were focusing on tumors, the effect of imaging time differences was expected to be very small.

## Conclusions

In conclusion, the pharmacokinetic parameters of DCE-MRI and total hemoglobin concentration measured by US-DOT were not well correlated. Although both were related to tumor angiogenesis, the contrast mechanisms used by these two modalities were different, and it was very difficult to match tissues in the analysis particularly given the heterogeneous nature of breast cancer. Nevertheless, the THC of US-DOT and K^*trans*^ of DCE-MRI were associated with parameters indicative of tumor aggressiveness with a high angiogenesis in breast cancer. Currently MRI is recommended for high-risk screening in Western countries, and it will be very interesting to see if US-DOT can serve as an alternative imaging modality with similar diagnostic performance compared to MRI. More studies are needed to establish the clinical value of US-DOT.

## Abbreviations

DCE: Dynamic Contrast-Enhanced
MRI: Magnetic Resonance Imaging
SER: Signal Enhancement Ratio
THC: Total Hemoglobin Concentration
TN: Triple-Negative
US-DOT: US-guided Diffuse Optical Tomography
